# Effects of intensive nutrition education and counseling on nutritional status of pregnant women in East Shoa Zone, Ethiopia

**DOI:** 10.3389/fnut.2023.1144709

**Published:** 2023-07-04

**Authors:** Ermias Bekele Wakwoya, Tefera Belachew, Tsinuel Girma

**Affiliations:** ^1^Department of Nutrition and Dietetics, Jimma University, Jimma, Ethiopia; ^2^Department of Pediatrics and Child Health, Jimma University, Jimma, Ethiopia

**Keywords:** nutrition, pregnancy, MUAC, RCT, Ethiopia

## Abstract

**Background:**

Nutritional status is defined as an individual’s health condition as it is influenced by the intake and utilization of nutrients. Maternal malnutrition is widespread throughout the world, with Sub-Saharan Africa and Asia bearing the brunt of the burden. The objective of this study was to evaluate the effect of intensive nutrition education and counseling on nutritional status during pregnancy.

**Methods and materials:**

The study was a one-year, two-arm parallel design cluster randomized controlled trial conducted in the East Shoa zone, Ethiopia, from January 1, 2021, to February 30, 2022. A total of 374 participants were enrolled in the intervention (*n* = 185) and control (*n* = 189) groups. End-line data were collected from 163 women, from each group. The intervention package provided three counseling sessions by trained midwives, three-page take-home brochures prepared in local languages, and the delivery of 18 weekly serial short text messages. The women in the control group received routine nutrition education from the health facilities. After adjusting for potential confounders, a linear mixed-effects model was employed to assess the intervention effect.

**Results:**

After the intervention, the mean mid-upper arm circumference in the intervention group increased by 1.8% (23.08 vs. 23.44, *p* < 0.01). Similarly, the proportion of undernutrition in the intervention group was 11% (25 vs. 36%, *p* = 0.02) lower compared to the control arm. At the end of the trial, women in the intervention arm had significantly better nutritional status than women in the control group (β = 0.47, *p* < 0.01).

**Conclusion:**

The findings showed that intensive nutrition education and counseling using the health belief model was effective in improving nutritional status and reducing undernutrition among pregnant women. As a result, nutrition education and counseling using HBM constructs, as well as regular reminder messages, should be provided to pregnant women as part of the routine antenatal care service.

## Introduction

Prenatal nutrition is a key component of healthy pregnancy outcomes (1). Improving nutritional status before and during pregnancy can reduce their risk of pregnancy complications such as preeclampsia, gestational diabetes, the risk of birth defects, intrauterine growth restriction, and later chronic disease (2). The prevalence of maternal malnutrition is high in the world, mainly in Sub-Saharan African and Asian countries (3, 4). In Ethiopia, maternal undernutrition is persistently high.

According to the Ethiopian Demographic and Health Survey (EDHS) report, 22% of sexually active women are undernourished (BMI less than 18.5) (5). Inconsistent with this report, studies carried out around the various regions of Ethiopia indicated that the proportion of maternal undernutrition is remarkably high. The prevalence of under nutrition ranged from 21.8 to 43.1% among pregnant women, with rural women having a greater prevalence. These studies were conducted at health institutions and community level (6–9).

Maternal anthropometric measurement and intake of optimum diet are determinants of fetal weight and gestational age at birth. Mid Upper Arm Circumference (MUAC) is the recommended measurement technique for nutritional status, because of its sensitivity in identifying undernutrition and its simplicity. It is also the preferable technique in low-resource settings where women have thin subcutaneous fat, changes in MUAC are more likely to reflect changes in muscle mass (10). Low maternal MUAC has been shown effective in identifying adverse birth outcomes such as preterm birth, intrauterine growth restriction, birth asphyxia, and small for gestational age. Therefore, MUAC measurement is recommended over pre-pregnancy weight to determine the risk of unfavorable pregnancy outcomes (11).

A woman’s pregnancy is an experience of life that can influence both her current health and the health of her developing fetus; it can also raise awareness of nutrition issues and change a woman’s dietary practices over time (11). Nielsen et al. reviews found that prenatal nutrition education and counseling treatments had a beneficial effect on pregnant adolescents’ diet quality and understanding of nutrition (12). Another systematic review of RCTs primarily conducted in high-income nations found that prenatal nutrition counseling decreased excessive gestational weight gain. The interventions included in these trials are; individual dietary consultation, group education, consultation with a dietician, and stepped-care behavioral interventions (13).

Gaetke et al. claim that even a single dietary counseling session with a registered dietician is associated with improved short-term clinical results (14). In the general population, it is suggested that nutrition education is a recommended sustainable and promising strategy to reduce maternal malnutrition (15). However, the studies done in Ethiopia show the routine nutrition education given to pregnant women by the health care system is vague and inconsistent (16). Because of this, child and mother malnutrition has continued to be a serious public health issue in the nation (5).

Compared to the period before preconception, pregnant women are more eager to know what they should eat and what not (17). Therefore, pregnancy is a good time for education. However, the effectiveness of counseling depends on the proper use of theories of behavioral science (18). Validated behavior change theories and models within the field of nutrition, provide systematic explanations for dietary behavior change that are integral to the nutrition care process, outcome evaluation, intervention, and guiding nutrition assessment. Health Belief Model (HBM) is the most widely used framework for health-related studies and has focused on disease prevention and preventive behaviors. HBM includes fundamental ideas that foretell why people take precautions to prevent getting sick. These constructs of HBM are perceived susceptibility, perceived severity, perceived benefits, perceived barriers, cues to action, and self-efficacy (19). A meta-analysis showed that the health belief model applies to many types of behavior across populations (20).

Although healthcare practitioners perceived nutrition education to be important, because of a barrier like shortage of time, inadequate space, poor counseling skills, and absence of documentation, women are not receiving adequate nutrition education during antenatal care (ANC) follow-up (21, 22). Furthermore, counseling was provided only once to the mothers throughout ANC follow-up, that is at their first visit only (23).

In the study area, there was a dearth of information on the effect of intensive nutrition counseling and education package on the nutritional status of expectant mothers. Therefore, the purpose of this study was to evaluate the effect of intensive nutrition education and counseling on nutritional status during pregnancy which was measured using MUAC. The study’s findings might help planners and policymakers at the national and local levels improve nutrition counseling practices.

## Materials and methods

### Study area

The study was carried out in East Shoa Zone, Oromia region, Ethiopia from January 4, 2021, to February 28, 2022. The total population in the East Shoa zone is 1,567,953 of which 48.3% were female. According to the East Shoa zone report of 2020, the healthcare facilities in the zone are providing antenatal care for more than 54,408 pregnant women per year. The research location and participants are fully discussed elsewhere (24).

### Study design and ethics

**FIGURE 1:**
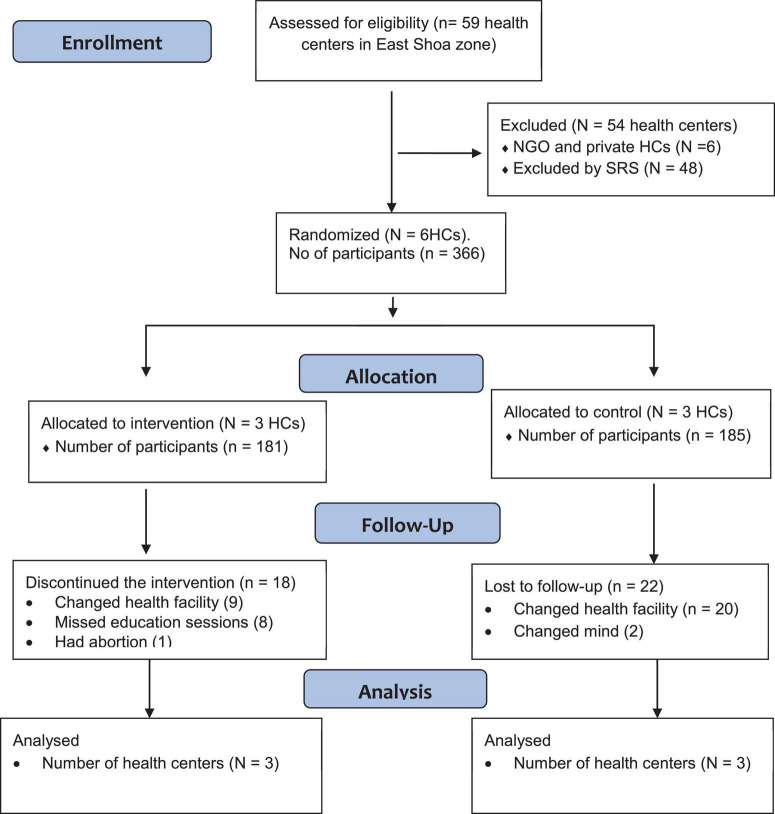
The flow of the study participants through the trial according to the criteria recommended in the CONSORT guideline. HCs, health centers; NGO, non-governmental organization; SRS, simple random sampling.

The study design was a two-arm parallel cluster randomized controlled trial. The Helsinki Declaration’s guiding principles and the standards of good clinical practice were followed during the study’s execution. The Institutional Review Board of Jimma University gave their approval to the research protocol (IHRPGD/S21/2018). Each study participant signed a written informed consent form, and pregnant women who could not read or write gave their fingerprints before the trial could begin. The study was registered on May 29, 2022, in the Iranian Registry of Clinical Trials (IRCT20220508054783N1). Results were reported by the Consolidated Standards of Reporting Trials (CONSORT) guideline (Figure 1) (25).

### Inclusion and exclusion criteria

Pregnant women were identified using women’s self-reports of pregnancy status confirmed by a urine HCG test. The study enrolled pregnant women before 16 weeks of gestational age. Pregnant women who refused to give verbal consent, who intended to leave the research area before delivery, or who had a mental illness or a chronic illness like hypertension or diabetes mellitus were excluded from the study. Those women who had a chronic illness like hypertension or diabetes mellitus were excluded, because these diseases were considered as a factor that can affect the nutritional status of pregnant women and could mask the true effect of nutrition counseling on the nutritional status of pregnant women.

### Sample size determination

Using G Power 3.1.9.2 and Fisher’s exact test with 80% power and 5% precision, the sample size was calculated. Kedir et al. reported that 24% of expectant mothers (P1) had undernutrition and assuming P1 and P2 have a 17% difference (26, 27).

The calculated sample size was increased by a design effect of two since cluster sampling was employed with a 15% loss to follow-up, the ultimate sample size for both arms was 374 pregnant women. As a result, 189 women in the control group and 185 women in the intervention group were enrolled in the trial, along with all pregnant women who met the inclusion criteria.

### Intervention allocation and randomization

From 59 health centers in the East Shoa zone, Non-governmental and private health centers were purposively excluded from the study. Six health centers were selected by simple random sampling (SRS) technique. Health centers with non-adjacent catchment areas were selected from each woreda by SRS (lottery) method. The six selected health centers (Clusters) were randomly allocated to the control and intervention arm by a 1:1 ratio. The cluster size in the control group was 63 study subjects in each cluster. Whereas, in the intervention arm, 61 participants were allocated to the first cluster and 62 participants were assigned to each second and third cluster.

Study participants were selected using systematic random sampling techniques following the Kth value. By dividing the total number of pregnant women who followed ANC during the study period by the sample size, the Kth value was calculated, and it was found to be five. Pregnant women who arrived first were counted as the first participants, and study participants who came at the fifth interval were also considered until the predetermined sample size was reached.

To minimize message contamination, cluster randomization was used. Pregnant mothers in the same health center were more likely to interact and discuss intervention messages. To prevent this, all pregnant women at one health center were enrolled in the same arm of the study (either in the control or intervention arms). Buffer zones (Non-adjusted catchment area health centers) were left between the control and intervention health centers (clusters) to prevent information contamination.

### Intervention

The intervention group received intensive nutrition education and counseling package (INECP) which had 3 components. These include, nutritional counseling was given in three sessions by trained midwives to pregnant women at the intervention arm, three-page brochures to take home in the local languages (Amharic and Afan Oromo) given to pregnant women at the intervention health center, 18 weekly serial short SMS text sent on mobile phone in local language was delivered to pregnant women in the intervention arm. In routine nutritional counseling professionals have been advising pregnant women only to eat one additional meal from available foodstuffs. However, in our study, the intervention given differs from the routine, for it contains intensive nutrition counseling sessions by trained healthcare providers on nutrition during pregnancy and counseling skills, delivery of leaflets containing core messages in local languages, and serial weekly SMS text on mobile phones.

The core nutrition message delivered during the counseling sessions, serial sms text, and delivered leaflet was promoting dietary diversity and additional meals during pregnancy, emphasizing an iron-rich meal, promoting sufficient protein and energy intake, promoting consistent use of the iron-folic acid tablet, use of iodized salt, counseling about healthy eating and reducing heavy workload, preventive deworming after the first trimester, regular antenatal care follow up and bed net use.

The training module was adapted from a blended and integrated nutrition learning module (BINLM). The core message was adapted from the WHO and Ethiopian Ministry of Health national module (27, 28).

The counselors and supervisors received a three-day intensive training that included role-playing using a training manual. As counselors and supervisors of the counseling process, respectively, six BSc midwives and two MSc nutritionists were hired. The training was facilitated by the principal investigator. Invited nutritionists who were certified by training of trainers (TOT) in BINLM supervised the training. The objective of the training was to introduce nutrition during pregnancy by addressing food groups category, expected weight gain, energy requirement, other food ingredients, lifestyle change, food safety issues, common problems during pregnancy, benefits of fulfilling nutrient requirements, and consequences of maternal malnutrition.

Aside from that, susceptibility to and severity of malnutrition in pregnant women and their fetus were included during counseling sessions. In the counseling manual, there was also information on the advantages of eating a sufficient number of diversified meals and the barrier that prevents one from taking a balanced diet.

Additionally, the training aimed to improve important counseling skills, and GALIDRAA steps to counseling (Greet, Ask, Listen, Identify, Discuss, Repeat, Agree, and Appoint) was addressed in depth (29). The health belief model was discussed in detail as a means of delivering nutrition education messages.

Role play, discussion, and power point presentations were used to support the training.

Additional resources, including modules and summary brochures, were also given to the trainees for display in their ANC room. Results of pre-post test questions provided to participants based on the training module were used to evaluate the training. The intervention arm health facilities received one-week follow-up supervision to assist identify and addressing any implementation-related challenges and fill up any training gaps.

Throughout each woman’s pregnancy, she went to three counseling sessions. Trained midwives provided individualized dietary counseling. During counseling, counselors employed a client-centered approach to determine the dietary habits and unique nutritional requirements of pregnant women. Counselors took into account the requirements of the women, their household income, and any barriers they found before allowing the women to select advice that was readily available, palatable, and cheap in their area.

A counseling guide containing the main topics was used, and the counseling sessions lasted 30 min over the first three appointments that followed. The initial counseling was provided before 16 weeks of gestation and concentrated on the use of iodized salt, basic nutrition, dietary categories, food selection, preparation, and frequency of meals. The second counseling session included the entirety of the counseling manual and was delivered during the second trimester of pregnancy. In the early third trimester of pregnancy, the last counseling session was provided.

Module, pamphlets, MUAC tape, and revised ANC logbook were provided to health centers in the intervention group. Take-home brochures of Amharic and Afan Oromo were given to study participants in the intervention arm. The pamphlets include clear and easy-to-follow core messages, actionable maternal recommendations in the form of bullet points, and an explanation of the many aspects of pregnancy nutrition.

Messages of the leaflet and serial short SMS text prepared in the local language delivered to the intervention group also incorporated the health belief model as a means of delivering nutrition messages. The health care system routinely provided nutrition education to the women in the control arm. Both the control and intervention arms’ pregnant women got access to additional ANC services.

### Data collection and measurement

The approaches used to obtain the required data were one-to-one interviewer-administered questionnaires and anthropometric measurements. The questionnaire was developed after a thorough review of the literature on the subject matter. The questionnaires included sociodemographic characteristics of women and their households, food security (30), obstetric history, HBM, MUAC, and nutrition knowledge.

Socio-demographic characteristics and obstetric history information were collected at the baseline. While data on food security, MUAC, and HBM were taken before and after the implementation of the intervention.

Six data collector midwives and 2 supervisors were trained for 3 days using a training guide centered on data collection procedures and tools. Questionnaires were initially written in English and translated to Afan Oromo and Amharic then back-translated to English by an expert fluent in English and the local languages. The tools were pre-tested before using for actual data collection. All data were obtained in the respondents’ mother tongue language. The interview takes 40 minutes on average to complete the questions. Data was collected in hard copy. Supervisors and principal investigator daily monitor the accuracy of the data. The privacy of the women was secured during the interview. The data collection procedure was monitored by the principal investigator and supervisors. A daily meeting was conducted by the data gathering and counseling team to go through any problems that came up.

Left mid-upper arm circumference (MUAC) was measured at the anatomic landmark of the midpoint between acromion and olecranon processes of the non-dominant hand by palm facing upward with flexing the women’s elbows to 90^0^. The measurements were taken twice by using inelastic MUAC tape and reading the measurement to the nearest 0.1 cm. Women with MUAC < 22 cm were considered undernourished, and ≥ 22 cm were considered well-nourished. The primary outcome of the study was the nutritional status of the pregnant women which was determined by measuring MUAC. The household food security status was assessed by using the Food Insecurity Access Scale (HFIAS) measurement tool (30).

The post-intervention data were collected between weeks 36 and 38 of pregnancy. Pregnant women who missed any counseling sessions were deemed to have “did not adhere” to the study’s standards, while those who withdrew from it were labeled as “lost to follow up”. 27 validated questions were used to measure the food security status (30). A household was classified as food secure, mildly, moderately, or severely food insecure if it possessed less than 2, 2–10, 11–17, and >17 food insecurity indicators, respectively. Those who scored above the mean value for knowledge questions were considered as knowledgeable and those who scored less than the mean value were considered as not knowledgeable. The Likert scale was used to score the perceived susceptibility, severity, benefit, and barriers of women. The responses were re-categorized such that those who answered ‘strongly agree’ or ‘agree’ were merged to ‘agree’ and those who responded ‘disagree’ or ‘strongly disagree’ were merged to ‘disagree’.

### Intervention fidelity

Based on the Health Behavioral Change Consortium recommendations the intervention given was assessed by a fidelity criteria checklist. The checklist includes intervention design assessment, counseling process, training of counselors, recipient of intervention, and implementation of the skills learned from the intervention (31, 32). This checklist was used by the principal investigator and supervisors and the core messages of the intervention were assessed.

The trial used the same number of clusters for both study arms. To prevent information contamination non-adjacent health centers (clusters) were selected. Pretest was done before the initiation of the experiment. A counseling guide and control group was employed in the trial. Every pregnant woman in the intervention arm received an equal number, length of contact, and frequency of the counseling session. For pregnant women who have no mobile phone, their partner or neighbor, or a nearby person’s contact number was used.

Counseling skill training was given using a training manual, and it includes a role-play and mock counseling practice. The training session has pre and post-training tests and practical evaluation. Process evaluation of the counseling session was done for randomly selected sessions by the process evaluator. Data collectors, counselors, and participants were all blinded to the study’s hypotheses. Until the analysis was finalized the data entry process was blinded by labeling the data with a unique number.

The counseling procedure was overall supervised by the principal investigator and supervisors. Intervention receipt of pregnant women was evaluated by awareness of the women on the core content of the message delivered by intervention through interviews.

### Data analysis

The quantitative data were coded and entered into Epidata V.3.1 to minimize design skipping patterns and logical errors. A cleaned copy of all the data was transported from Epidata to SPSS version 26 for cleaning, editing, and data processing.

The baseline socio-demographic characteristics of the women were compiled using descriptive statistics according to group status. To compare the baseline characteristics of the intervention and control groups, a chi-square test was used.

The wealth index was assessed using principal component analysis (PCA) by considering fixed assets such as the source of drinking water, possession of television, radio, mobile phone, availability of a separate kitchen from the living house, household assets, livestock, and agricultural land ownership. These variables were dichotomized and coded ‘1’ for the household possessing the asset and ‘0’ for the rest. Factor scores were produced using variables having a commonality value of greater than 0.5 in PCA. Finally, the factor scores ranked ordered into three relative measures of socioeconomic classes (poor, medium, and rich).

Independent samples and paired sample t-tests, respectively, were used to compare the MUAC between and within the control and intervention groups. In all analyses, a two-sided *p*-value of < 0.05 was used as a cutoff point to declare statistical significance. Multicollinearity between the independent variables was assessed by using variance inflation factors (VIF > 10 was considered as the existence of collinearity).

A per-protocol analysis was performed in this trial. The final analysis comprised pregnant women who participated in three counseling sessions and provided end-line information. The effects of the intervention on changes in the nutritional status of pregnant women over time were assessed using a linear mixed-effects model. This model allowed us to account for the correlation of findings, due to the repeated measurements (pre- and post-intervention) and the grouping of individuals within the six randomly selected clusters.

Participants and clusters were analyzed as random effects during model fitting. Additionally, this approach makes it possible to manage the impact of various confounding factors (food security, wealth index, education, family size, and age). The average nutritional status across all clusters varied by 0.004 according to the intercept-only model, which calculates the variance of the cluster-level residual errors as 0.004.

The intra-cluster correlation coefficient was (0.001) which showed that no need to fit a third-level model. The two-level model was therefore fitted to take into consideration time-invariant variables at the individual level.

The difference in difference was used to estimate the effect of intervention by comparing the changes in outcomes over time between the control and intervention groups. The effect of the intervention was assessed by testing the interaction term between time and treatment allocation. The model estimate data on outcomes, treatment allocation, and periods. The coefficient of the interaction term was used as an estimate of the treatment effect. The SPSS software version 26 was used to conduct all statistical analyses.

## Results

### Response rate and attendance

A total of 366 (89.07%) participants were surveyed at the baseline. All participants in the intervention and control groups attended the first session. 18 (11.0%) from intervention group discontinued the intervention and 22 (13.5%) form control group were lost from the follow up. A total of 326 from both arms were fit for final analyses. The overall follow-up of study participants through the trial was summarized by the CONSORT guideline flow chart (Figure 1).

### Socio-demographic characteristics

A total of 366 eligible pregnant women were recruited to the study from six health centers. Of these, 326 (Intervention = 163, Control = 163) women who strictly adhered to the protocol were included in the final analysis with an 89.1% retention rate. The mean ± SD age of the participants in the intervention and control groups were 24.78 (± 4.3) and 26.8 (± 4.46), respectively. Most participants in the intervention (87.7%) and control arm (86.5%) were between the 20–24 age groups.

The majority of the study participants in the intervention (60.7%) and control (73.0%) groups were housewives. The socio-demographical characteristics of the participants which are known to affect the nutritional status at baseline had no statically significant difference between the two arms (*P* > 0.05) (Table 1).

**TABLE 1:** The baseline characteristics of pregnant women.

Variables	Intervention group (*N* = 163)		Control group (*N* = 163)		
	Frequency	(%)	Frequency	(%)	*P*
Number of clusters	3		3	
**Age (years)**					
<20	16	9.8	14	8.6	0.23
20–34	143	87.7	141	86.5	
≥35	4	2.5	8	4.9	
**Religion**					
Orthodox	81	49.7	76	46.6	0.1
Muslim	48	29.4	48	29.4	
Protestant	27	16.6	36	22.1	
Others	7	4.3	3	1.8	
**Educational status**					
Unable to read and write	32	19.6	33	20.2	0.11
Read and write	36	22.1	26	15.9	
Primary (1–8)	56	34.3	52	31.9	
Secondary (9–12)	26	15.9	33	20.2	
Tertiary (college and above)	13	7.9	19	11.7	
**Husband educational status**					
Unable to read and write	14	8.8	14	8.6	0.705
Read and write	36	22.5	32	19.8	
Primary (1–8)	39	24.4	45	27.8	
Secondary (9–12)	41	25.6	43	26.5	
Tertiary (college and above)	30	18.7	28	17.3	
**Occupation**					
Housewife	99	60.7	119	73	0.08
Government employee	17	10.4	12	7.4	
Private employee	32	19.6	16	9.8	
Daily laborer	5	3.1	5	3.1	
Merchant	5	3.1	9	5.5	
Others	5	3.1	2	1.2	
**Marital status**					
Married	160	98.2	162	99.4	0.42
Others	3	1.8	1	0.6	
**Wealth index quartile**					
Poorest	19	11.7	16	9.8	0.88
Poor	20	10.4	17	10.4	
Middle	101	52.3	107	65.7	
Richer	23	11.9	23	14.1	

### Health belief model score before and after intervention and their correlation with MUAC

At baseline, there was no significant difference between the HBM constructs score of the intervention and control groups. Perceived susceptibility and perceived benefit were significantly improved among the intervention group. The mean nutrition knowledge score significantly increased from 7.85 to 8.12 (*p* < 0.01) in the intervention group (Table 2). Perceived benefit, perceived susceptibility, and nutrition knowledge had a positive significant correlation with MUAC of pregnant women (Table 3).

**TABLE 2:** Health belief model construct score before and after intervention among pregnant women in East Shoa zone, central Ethiopia.

HBM constructs	Time	HBM construct scores		*p*-value
		Intervention (*N* = 163)	Control (*N* = 163)	
Perceived susceptibility	Baseline	3.75 (±0.93)	3.62 (±0.88)	0.33
	Endline	4.04 (±0.95)	3.65 (±0.88)	<0.01
	*P*-value	<0.01	0.79	
Perceived severity	Baseline	3.76 (±1.05)	3.57 (±1.16)	0.12
	Endline	3.85 (±1.06)	3.53 (±1.15)	0.01
	*P*-value	<0.01	0.25	
Perceived benefit	Baseline	4.09 (±0.91)	3.96 (±1.03)	0.28
	Endline	4.21 (±0.94)	3.97 (±0.97)	0.03
	*P*-value	<0.01	0.85	
Perceived barrier	Baseline	4.03 (±0.89)	4.11 (±0.93)	0.41
	Endline	4.10 (±0.91)	4.19 (±0.95)	0.40
	*P*-value	0.43	0.46	
Nutrition knowledge	Baseline	7.85 (±1.31)	7.61 (±1.63)	0.11
	Endline	8.12 (±1.25)	7.69 (±1.64)	<0.01
	*P*-value	<0.01	0.07	

**TABLE 3:** Correlation of health belief model constructs with nutrition knowledge and MUAC of pregnant women in East Shoa zone, Central Ethiopia.

Variables	Intervention	Perceived susceptibility	Perceived severity	Perceived benefit	Perceived barrier	Nutritional knowledge	MUAC
Intervention	1						
Perceived susceptibility	0.15 0.05	1					
Perceived severity	0.05 0.39	–0.12 0.32	1				
Perceived benefit	0.14 0.01	0.16 0.00	–0.06 0.31	1			
Perceived barrier	0.14 0.79	0.05 0.41	0.08 0.12	–0.04 0.51	1		
Nutritional knowledge	0.18 0.03	0.29 0.00	0.09 0.08	0.24 0.00	–0.44 0.43	1	
MUAC	0.52 0.00	0.46 0.00	0.15 0.07	0.33 0.00	0.01 0.83	0.33 0.00	1

### Nutritional status of pregnant women

Before the educational intervention, there was no significant difference in the mean MUAC scores of the women enrolled in the intervention and control groups. (23.08 ± 1.56 Vs 23.26 ± 1.60, *p* = 0.07) (Table 4). The intervention and control groups had a comparable proportion of undernutrition at baseline (28 Vs 32%, *p* = 0.55). There was a significant change in both nutritional status and proportion of undernutrition between the intervention and control groups after the intervention. The mean MUAC difference before and after the intervention was 0.36, *p* < 0.01 [23.08 (1.56) Vs 23.44 (1.60)]. The proportion of undernutrition among pregnant women in the intervention group was lower by 11% (25 Vs 36%) as compared to the control group with a *p*-value *p* = 0.023. The T-test result showed that the intervention improves the mean MUAC by 1.8% (Table 5).

**TABLE 4:** Comparisons of mean MUAC scores of pregnant women in the experimental and control groups before and after education intervention in East Shoa zone, central Ethiopia, 2021.

Groups	Baseline^1^ MUAC (cm)	End line MUAC (cm)	Difference	*p*-value^2^
Intervention	23.08 (1.56)	23.44 (1.60)	0.36 (1.23)	<0.01
Control	23.26 (1.63)	23.10 (1.67)	–0.15 (1.01)	0.06
*p*-value^3^	0.07	0.03	<0.01	

^1^Data are mean ± SD.

^2^Paired *t*-test.

^3^Independent *t*-test.

**TABLE 5:** Differences between baseline and endline measurements of MUAC and difference of the differences between the intervention and control groups, East Shoa zone, Central Ethiopia, 2021.

Variable	MUAC difference in Intervention^1^ (cm)	MUAC difference in control (cm)	Difference (cm)	*p*-value
MUAC	0.36 (1.23)	–0.15 (1.01)	0.49 (1.23)	<0.01

^1^Data are mean ± SD, linear logistic regression.

### Effect of nutrition education on the nutritional status of pregnant women

The variability of average MUAC across individuals was 2.68 and statistically significant (*p* < 0.05). In this study, since the intra-cluster correlation coefficient was 0.02, two-level models were fitted (Table 6). After controlling for food security, family size, educational status, women’s decision-making, and wealth women in the intervention group showed significantly improved nutrition status at the end of the study trial (β = 0.50, *p* < 0.01).

**TABLE 6:** Linear mixed effect model predicting the nutritional status of pregnant women in East Shoa zone, Central Ethiopia.

Fixed effect variables	Model 1		Model 2		Model 3	
	Estimate (SE)	95% CI	Estimate (SE)	95% CI	Estimate (SE)	95% CI
Intercepts	**23.40 (0.15)**	23.1–23.7	**23.49 (0.15)**	23.20, 23.79	**23.70 (0.28)**	23.13, 24.27
Intervention effect			**0.46 (0.08**)	0.29, 0.63	**0.47 (0.08)**	0.31, 0.64
Endline control			–0.02 (0.03)	–0.09, 0.48	–0.06 (0.09)	–0.25, 0.12
Baseline intervention			**0.82 (0.03)**	0.76, 0.88	0.9 (0.07)	0.98, 1.01
Food security					0.04 (0.34)	–0.64, 0.73
Educational status					0.38 (0.35)	–0.29, 1.07
Wealth					0.36 (0.50)	–0.62, 1.35
Women decision making power					–0.16 (0.35)	–0.86, 0.53
Family size					0.27 (0.48)	–0.67, 1.22
**Random effect**						
Variances	3.49 (0.27)		2.92 (1.71)		2.70 (1.6)	
ICC	0.02		0.01		0.03	
AIC	1341.86		645.53		702.87	
Parameters	3		6		14	

ICC, intraclustor correlation coefficient; AIC, Akaike information criteria. The bold values represent the statistical significance at *p* ≤ 0.05.

The variation of the average nutritional status across all clusters was 0.0035, which was not statistically significant (*p* = 0.90), and the intercept-only model predicts the variance of the cluster-level residual errors as 0.0026. The intra-cluster correlation coefficient was closer to zero (0.001), indicating that no third-level model needed to be fitted.

## Discussion

The study assessed the effect of intensive nutrition education and counseling during antenatal care on the nutritional status of pregnant mothers attending ANC service in selected interventional areas. The primary outcome of this study was nutritional status which was measured by MUAC. The study has compared the effect of the intervention with routine nutrition education given by the existing healthcare system.

In the current study, perceived susceptibility and perceived benefit were significantly improved and were also significantly correlated with the MUAC of pregnant women after the intervention. However, perceived severity and perceived barrier were neither significantly improved nor significantly correlated with MUAC of pregnant women after the intervention. According to the report from northeast Ethiopia, except perceived barrier, all HBM constructs were significantly improved among pregnant women in the intervention group (33). The reason that perceived severity was not improved in the current study might be due to poor emphasis given to the adverse effects caused by undernutrition in society. On the other hand, prior studies have shown that perceived severity was less often associated with the desired health behavior whereas, perceived susceptibility and benefit were consistently associated with the desired health behavior (34).

In the present study, nutrition knowledge was significantly improved in the intervention group and also significantly correlated with the MUAC of pregnant women in the intervention group. This finding is in line with the study done in southwest Ethiopia where the mean nutrition knowledge score has significantly increased from 6.9 to 13.4 following nutrition education and counseling intervention (35). One of the key advantages of nutrition education is improving the knowledge of women on optimal diet during pregnancy, thereby positively influencing the attitude and practice towards good nutrition (36–38). Nutrition education and counseling have been shown to have a positive effect on the awareness of expectant mothers about important micronutrient intake and the negative impact of food aversion during pregnancy (39, 40). Integration of nutrition education and counseling supplemented with mobile health during antenatal care should get emphasis to improve the nutritional status of pregnant women and thereby their nutritional status.

In this study, the socio-demographic characteristics of participants in both groups were not statistically different. The nutritional status of pregnant women in control and intervention groups was also similar at the baseline. At the end line, the nutritional status of pregnant women who received at least three counseling sessions and weekly serial SMS mobile phone text significantly increased by 36% after the nutritional education intervention (*P* < 0.001). This finding is in line with the study done in Northwest Ethiopia where guided nutrition counseling given to pregnant women using the health belief model and theory of planned behavior improved the nutritional status during pregnancy (β = 0.615, *p* = < 0.001) (41).

The prevalence of undernutrition among pregnant women in the control group has risen relative to the baseline, which is consistent with findings from earlier studies in Ethiopia (23.26 vs 23.10) (41). Whereas, the prevalence of under-nutrition in the intervention arm was 11% lower than the control group (25 vs. 36%) with a *p*-value of 0.023. The finding is consistent with the study done in India that showed positive effects in improving the weight gain and nutritional status of pregnant women after delivering nutrition core messages using individual counseling, weekly home visits, and group meetings (42).

In this study after adjusting for the educational status of pregnant women, food security, women’s decision-making power, family size, and wealth, pregnant women in the intervention group showed a significant improvement in nutritional status at the end of the trial. Prior studies have also indicated that nutrition education interventions have improved the nutritional status of pregnant women by improving their dietary practices and improve weight gain during pregnancy (35, 41). The nutrition education intervention can improve the nutritional status by improving the knowledge of women on appropriate diets during pregnancy. The improved knowledge helps them to start good dietary practices and this in turn helps them to have a good nutritional status (43).

Although the study was done in multiple health centers and a well-designed randomized controlled trial, there are still limitations. This study was done among pregnant women who came to the selected health centers and this made the result of this study impossible to generalize for the pregnant women living in the community, who were not attending antenatal care at these health facilities. Furthermore, all of the responses, except the MUAC measurement, were based on the women’s self-report, recall, and honesty in responding to the questions.

## Conclusion

This study revealed that intensive nutrition education and counseling using the health belief model was effective in improving the nutritional status and minimizing the level of undernutrition among pregnant women. Counseling by using HBM, regular SMS, and leaflets was found to be important in improving the nutritional status of pregnant women in this study area. Therefore, nutrition education and counseling by using HBM constructs and regular reminder messages have to be provided to pregnant women as part of the regular antenatal care service.

## Data availability statement

The raw data supporting the conclusions of this article will be made available by the authors, without undue reservation.

## Ethics statement

The studies involving human participants were reviewed and approved by the Institutional Review Board of Jimma University (protocol number: IHRPGD/S21/2018), Jimma, Ethiopia. The patients/participants provided their written informed consent to participate in this study.

## Author contributions

EW, TB, and TG involved in conceptualization, proposal development, questioner formulation, ethical clearance, registration of clinical trial, supervision in data collection, intervention and follow up, data clearance, data analysis, writing up, and manuscript preparation. All authors contributed to the article and approved the submitted version.
